# Integrating Resistant and Pulsatile Right Ventricular Load Improves Severity Assessment in Heart Failure Patients

**DOI:** 10.1016/j.jacadv.2026.102838

**Published:** 2026-06-17

**Authors:** Odd Bech-Hanssen, Thomas Lindow, Marco Astengo, Tomas Mellberg, Entela Bollano, Göran Rådegran

**Affiliations:** a Departments of Clinical Physiology; b Cardiology, Sahlgrenska University Hospital, Gothenburg, Sweden; c Institute of Medicine, The Sahlgrenska Academy at the University of Gothenburg, Sweden; d Respiratory Medicine, Allergology, and Palliative Medicine, Clinical Sciences, Lund University, Lund, Sweden; e Department of Medicine, Department of Research and Development, Växjö Central Hospital, Region Kronoberg, Växjö, Sweden; f Department of Clinical Sciences Lund, The Section for Cardiology, Lund University, Lund, Sweden; g The Haemodynamic Lab, The Section for Heart Failure and Valvular Disease, VO. Heart and Lung Medicine, Skåne University Hospital, Lund, Sweden

**Keywords:** left heart disease, prognostication, right heart catheterization, right ventricular dysfunction, severity assessment

## Abstract

**Background:**

The 2022 European Society of Cardiology/European Respiratory Society (ESC/ERS) pulmonary hypertension definition only consider resistant right ventricular (RV) load (pulmonary vascular resistance [PVR]), but not pulsatile load (pulmonary artery compliance [PAC]) known to be important.

**Objectives:**

The authors aimed to compare severity-grading of RV afterload using the current pulmonary hypertension definition with a novel RV afterload score and investigate the PVR-PAC relation in patients with heart failure.

**Methods:**

We included patients (n = 328) with reduced left ventricular ejection fraction (<50%) consecutively referred for right heart catheterization. For PVR and PAC separately, we allocated points for low (0 points), intermediate (1 point), and high (2 points) afterload. After summing the points, 0, 1 to 2, and 3 to 4 defined low, intermediate, and high RV afterload. The primary endpoint was all-cause mortality or a left ventricular assist device implantation.

**Results:**

The RV afterload score was superior in severity stratification compared with ESC/ERS phenotypes with stepwise significant differences (*P* < 0.001) in RV function, cardiac index, pulmonary artery wedge pressure, and prognostic value (*P* = 0.010). Compared with a low afterload score, the adjusted HR (95% CI) for a high score was 4.42 (1.86-10.6). The hyperbolic PVR-PAC relation (R^2^ = 0.48), allowed definition of 3 PVR zones: ≤2 WU with 56% low and 44% intermediate, 2.1 to 3.9 WU with 67% intermediate and 33% high load, and ≥4 WU with 100% high afterload score.

**Conclusions:**

We describe a novel RV afterload score that improves severity grading and prognostication compared with current ESC/ERS phenotypes and define PVR-intervals that might be helpful for therapy individualization and in upcoming trials with new therapeutic targets.

Pulmonary hypertension (PH) is a common finding in patients with left heart disease. Increased left ventricular (LV) filling pressure at rest or during exercise increases the pulmonary artery (PA) pressure and the right ventricular (RV) afterload. RV dysfunction in patients with left heart disease and the observed negative prognostic impact can, in most cases, be explained by the increased RV afterload and, to a lesser extent, by intrinsic RV myocardial dysfunction.^1^ In previous and current European Society of Cardiology/European Respiratory Society (ESC/ERS) guidelines, the definition of PH is based on the level of mean PA pressure and further subdivided according to the level of PA wedge pressure and pulmonary vascular resistance (PVR).^2^ Two different phenotypes with postcapillary PH in patients with left heart disease and PH has been described: isolated postcapillary PH (IpcPH) with increased PA wedge pressure and normal PVR and combined pre- and postcapillary PH (CpcPH) with increased PA wedge pressure and PVR. Recently, the ESC/ERS 2022 guidelines lowered the threshold for increased PVR from ≥3 WU to >2 WU. This change in definition will not affect the overall RV afterload burden in patients with left heart disease and heart failure, but changing the PVR threshold will consequently reclassify patients with postcapillary PH from IpcPH to CpcPH, and, importantly, it will lower the total RV afterload burden in patients with CpcPH. To what extent this changes the hemodynamic and prognostic profiles of IpcPH and CpcPH, respectively, is not well studied.

The guideline hemodynamic definition of PH only considers the resistive RV load (PVR). In PH, pulsatile loading (PA compliance [PAC]) is known to be another important determinant of the RV afterload^3^ and prognosis.^4^, ^5^, ^6^ In the pulmonary circulation, there is a strict inverse relation between PVR and PAC, the product being constant, allowing calculation of one from the other.^7^ However, in patients with left heart disease, that is opposed to the other subgroups of PH, the presence of increased PA wedge pressure is known to reduce PAC.^3^ Therefore, in patients with left heart disease associated PH, individuals with identical PVR may exhibit different PAC values depending on the level of PA wedge pressure, resulting in different RV afterload. The relation between PAC and PVR will change in the individual patient over time in a continuous manner with disease progression. In a patient with compensated left heart disease without PH or elevated PA wedge pressure both PAC and PVR will typically be normal. Initially, with development of decompensation and increased PA wedge pressure the pulsatile load will increase as PAC worsens, while the resistant load (PVR) remains normal. With further disease progression, there will be increased arterial vascular tone, arteriolar and venous remodeling with increased resistant load. The different pathophysiological mechanisms (LV backward failure, increased vascular resistance, and decreased compliance) involved in patients with left heart disease and PH are potential therapeutic targets. It follows that for a more comprehensive assessment of the RV afterload, that may facilitate individualized heart failure therapy, we need to consider not only the resistive load but also the pulsatile load. In the present study, we present a novel RV afterload score that combines pulsatile (PAC) and resistant (PVR) load. Each component of the RV afterload (PVR and PAC) was categorized into 3 levels (low, intermediate, and high), which were subsequently combined into an overall RV afterload score with the same 3 level classification.

The aims of the present study, including patients with left heart disease and reduced LV ejection fraction (<50%) were to compare grading severity of RV afterload using the current PH definition with a novel RV afterload score, and to investigate the PVR-PAC relation.

## Methods

### Study population

In this retrospective study, 364 patients, with left heart disease who underwent right heart catheterization at the Sahlgrenska University Hospital, Gothenburg, Sweden, between 2009 and 2019, were screened. All patients were considered to have left heart disease after a comprehensive diagnostic workup including right heart catheterization and coronary angiography. The main indications for catheterization were assessment of hemodynamic status as a part of a pretransplant workup, assessment of PH or restrictive cardiac physiology, and hemodynamic evaluation at the time of diagnostic endomyocardial biopsy. Patients with LV ejection fraction ≥50% (n = 24) and missing data at right heart catheterization (n = 12) were excluded. The final study population comprised 328 individuals. Of these, 289 (89%) underwent echocardiography within a week and 188 (57%) ergospirometry.

A retrospective chart review was performed to analyze clinical characteristics of all the patients. Blood samples obtained were analyzed by the Central Laboratory of Sahlgrenska University Hospital (accredited according to the EN ISO 15189:2022). The study protocol was approved by the Regional Ethical Review Board (Dnr. 286-18).

### Hemodynamic measurements

The period of fasting prior to the investigation was at least 4 hours and liquids stopped at least 2 hours before the procedure. We aimed to minimize the effect of fasting by keeping this period as short as possible without compromising the safety aspects. At catheterization there were no patients on temporary mechanical assist or intravenous inotropic medication. A PA catheter (7-F, Baxter Healthcare, Edwards Critical Care Division) was introduced using the Seldinger technique. In most cases, the internal jugular vein was used for access.

The following variables were measured or derived: heart rate, right atrial pressure, RV end-diastolic pressure, systolic PA pressure, diastolic PA pressure, mean PA pressure, PA wedge pressure, cardiac output, and PVR. PA wedge pressure was measured as the mean pressure during quiet breathing. Cardiac output was determined by the thermodilution method as the mean of 3 to 5 consecutive measurements not varying by more than 10%. Stroke volume and cardiac output were indexed to body surface area yielding stroke volume index and cardiac index. PVR was calculated as the difference between mean PA pressure and PA wedge pressure divided by cardiac output and expressed in WU. The transpulmonary pressure gradient was calculated as the difference between the mean PA pressure and PA wedge pressure. PAC and PA effective elastance were defined as stroke volume divided by pulmonary pulse pressure (systolic PA pressure − diastolic PA pressure) and systolic PA pressure/stroke volume, respectively. The RV stroke work index was defined as: stroke volume index × (mean PA pressure − right atrial pressure) × 0.0136. Reduced PAC was defined as ≤3.8 mL/mm Hg.^8^ The patients were divided according to hemodynamic PH phenotype into 3 groups: patients without PH (no PH, mean PA pressure ≤20 mm Hg), patients with IpcPH and CpcPH according to the 2022 ESC/ERS definition (IpcPH: mean PA pressure >20 mm Hg, PA wedge pressure >15 mm Hg, and PVR ≤2 WU; CpcPH: mean PA pressure >20 mm Hg, PA wedge pressure >15 mm Hg, and PVR >2 WU), and the 2018 World Symposium on Pulmonary Hypertension (WSPH) definition (IpcPH: mean PA pressure >20 mm Hg, PA wedge pressure >15 mm Hg, and PVR <3 WU; CpcPH: mean PA pressure >20 mm Hg, PA wedge pressure >15 mm Hg, and PVR >3 WU).^2^^,^^9^

### RV afterload score

For the RV afterload score, we aimed at defining 3 levels: low, intermediate, and high. To obtain this, we defined the 3 levels for PAC and PVR indicating low, intermediate, and high pulsatile and resistant load, respectively. The lower borders of the intermediate level were defined by what is considered normal, that is for PAC ≥3.8 mL/mm Hg^8^ and for PVR ≤2.0 WU.^10^ The lower borders of high pulsatile and resistant load were defined from previous studies on prognostic impact in patients with left heart disease. For high pulsatile load, that is, PAC ≤2.0 mL/mm Hg;^5^^,^^6^ for high resistant load, PVR ≥4 WU.^5^ Low, intermediate, and high pulsatile and resistant load were denoted 0, 1, and 2 points, respectively. By combining the PVR and PAC points, the maximum score is 4 points. Low, intermediate, and high RV afterload score was then defined as 0, 1 to 2, and 3 to 4 points, respectively (Supplemental Table 1).

### Clinical outcomes

Long-term mechanical circulatory support has replaced urgent transplantation, in patients with hemodynamically unstable heart failure or in those at considerable risk of deterioration during the waiting time for heart transplantation. Consequently, a composite of all-cause death and implantation of a LV assist device (LVAD) was chosen as the endpoint.

### Statistical analysis

Continuous variables are expressed as the mean ± SD, median (IQR), or as numbers (percentages). The association between hemodynamic phenotype, based on the 2018 and 2022 classifications, and the RV afterload score with the composite endpoint of all-cause mortality or implantation of a LVAD was analyzed using Cox proportional hazards regression. Results are reported with HRs with 95% CIs. Model discrimination is described with Harrell’s C-statistics and compared between models using a variance-based test for correlated C-statistics. Variables were considered confounders if, based on subject knowledge, they met the following criteria: 1) presumed association with the outcome; 2) presumed association with or uneven distribution across PH classes; and 3) not an effect of hemodynamic PH classes, that is, not on the causal pathway. Based on these criteria, age, sex, LV ejection fraction, presence of moderate/severe mitral regurgitation, and N-terminal pro–B-type natriuretic peptide (as a surrogate for elevated LV end-diastolic pressure) were included in the multivariable analysis. Time-to-event was calculated from right heart catheterization to death or LVAD implantation, whichever occurred first, or censoring. Patients were censored either at the end of follow-up that was 21st October 2025, or at the time of heart transplantation. Since pulmonary hemodynamics are expected to progress over time among patients with advanced heart failure, baseline classes are likely to become less representative with longer follow-up. Therefore, follow-up was truncated at 36 months. As a sensitivity analysis, Cox regression was also performed using the full follow-up time as well and the results are presented in the Supplemental Appendix. The assumption of proportional hazards was confirmed using Schoenfeld’s residuals. The incremental prognostic value of the RV afterload score to the 2022 ESC/ERS definition was evaluated by the likelihood ratio test in which a model with the 2022 ESC/ERS definition alone and a model with the 2022 ESC/ERS definition and the RV afterload score were compared. The prognostic value of adding PAC information to PVR was studied using the likelihood ratio test when comparing a model including PVR alone with a model with PVR and PAC.

The statistical analysis was performed using SPSS for Macintosh, version 28, and R version 4.3.1.

## Results

The mean age was 53 ± 13 years and 76.2% were males. A total of 208 patients (63%) had dilated cardiomyopathy, and 73 patients (22%) had ischemic heart disease. Right heart catheterization was performed in 210 patients (64%) as part of heart transplant workup, in 108 patients (33%) for assessment of PH or restrictive cardiac physiology, and in 9 patients (3%) in connection with an endomyocardial biopsy. Table 1 describes demographic and clinical data for the study population. A total of 289 patients (88%) had echocardiography ≤7 days preceding or following right heart catheterization. Echocardiography and right heart catheterization were performed during the same day in 64 patients (22%), the preceding or following day in 146 patients (51%), and within 48 hours in 54 patients (19%).

**Table 1:** Demographic and Clinical Data

Demographics	
Age (y)	53 ± 13
Male	76
Body mass index (kg/m^2^)	27 ± 4.7
Diagnosis	
Dilated cardiomyopathy	208 (63)
Ischemic heart disease	73 (22)
Cardiac sarcoidosis	10 (3)
Myocarditis	10 (3)
Cardiac amyloidosis	9 (2.7)
Hypertrophic cardiomyopathy	9 (2.7)
Valvular disease	4 (1.2)
Restrictive cardiomyopathy	3 (0.9)
Congenital heart disease	2 (0.6)
Comorbidities	
Diabetes	46 (14)
Hypertension	32 (10)
Previous cardiac surgery	25 (8)
Renal failure	16 (5)
Sleep apnea	10 (3)
Chronic obstructive pulmonary disease	10 (3)
History of pulmonary embolism	5 (1.5)
Medications	
Beta-blocker	306 (93)
Loop-diuretic	246 (76)
Aldosterone inhibitor	208 (63)
Angiotensin-converting enzyme inhibitor	179 (55)
Angiotensin 2 inhibitor	74 (23)

Values are mean ± SD or n (%).

### The 2022 ESC/ERS versus 2018 WSPH PH definition

**Figure 1:**
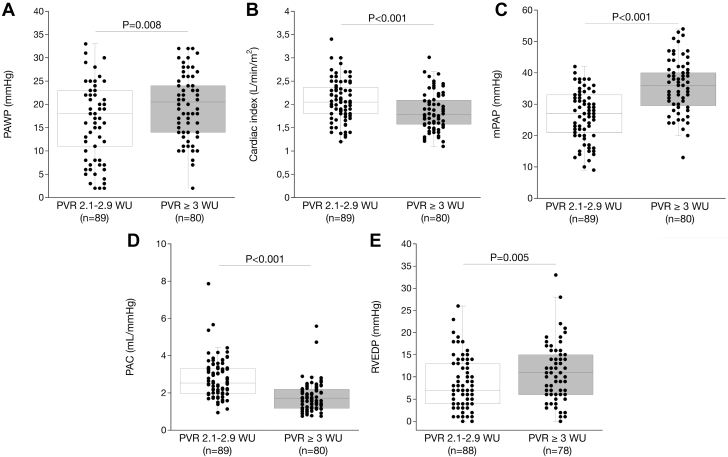
**Comparison Between Patients With Pulmonary Vascular Resistance 2.1 to 2.9 WU and Pulmonary Vascular Resistance ≥3 WU** Boxplots showing: (A) pulmonary artery wedge pressure, (B) cardiac index, (C) mean pulmonary artery pressure, (D) pulmonary artery compliance, and (E) right ventricular end-diastolic pressure (right ventricular end-diastolic pressure). mPAP = mean pulmonary artery pressure; PAC = pulmonary artery compliance; PAWP = pulmonary artery wedge pressure; PVR = pulmonary vascular resistance; RVEDP = right ventricular end-diastolic pressure.

Using the 2022 ESC/ERS definition increased the number with CpcPH from 78 to 146 and correspondingly reduced the number with IpcPH from 141 to 73 (Central Illustration). The prevalence of CpcPH increased by 55%. Patients with PVR ≥3 WU had compared with patients with PVR 2.1 to 2.9 WU more severe LV dysfunction with higher PA wedge pressure and lower cardiac index, more pronounced PH, lower PA compliance, and more severe RV dysfunction with higher RV end-diastolic pressure (Figure 1).

### RV afterload score

**Figure 2:**
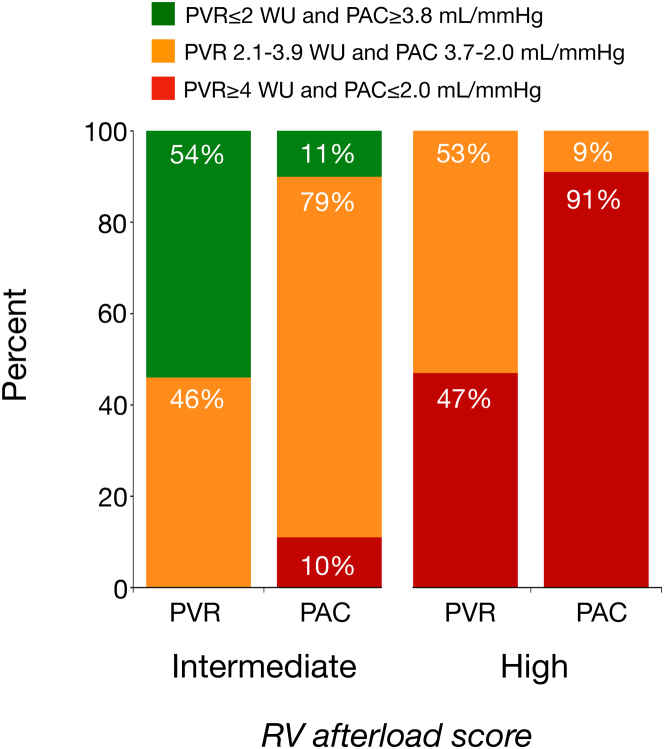
**Contribution of Resistive and Pulsatile Load in Right Ventricular Afterload Score** Distribution of low, intermediate and high levels of resistive, and pulsatile load in patients with intermediate and high right ventricular afterload score. PAC = pulmonary artery compliance; PVR = pulmonary vascular resistance; RV = right ventricle/ventricular.

**Figure 3:**
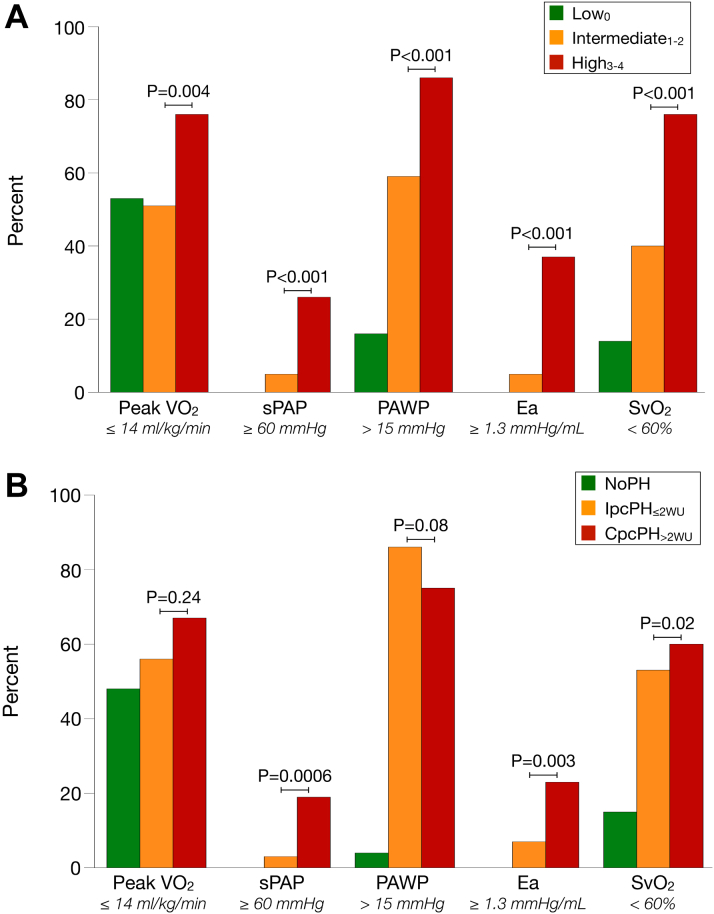
**Severity Grading Using Right Ventricular Afterload Score and 2022 European Society of Cardiology/European Respiratory Society Phenotype** The relation between increments of right ventricular afterload score (A) and the 2022 European Society of Cardiology/European Respiratory Society pulmonary hypertension phenotype (B) and the proportion with severely reduced functional capacity (peak maximum rate of oxygen consumption [VO_2_] ≤14 mL/kg/min), severe pulmonary hypertension (systolic pulmonary artery pressure ≥60 mm Hg), overt left ventricular decompensation (pulmonary artery wedge pressure >15 mm Hg, SvO2 <60%), and severely reduced pulmonary artery effective elastance (≥1.3 mm Hg/mL). CpcPH = combined pre- and postcapillary pulmonary hypertension; Ea = pulmonary artery effective elastance; IpcPH = isolated postcapillary pulmonary hypertension; NoPH = no pulmonary hypertension; PAWP = pulmonary artery wedge pressure; sPAP = systolic pulmonary artery pressure; SvO_2_ = mixed venous oxygen saturation.

A total of 89 patients (27%) had 0 points, indicating low RV afterload, 147 patients (45%) had 1 or 2 points indicating intermediate RV afterload, and 92 patients (28%) had 3 or 4 points indicating high RV afterload. Pulsatile loading with reduced PAC was present in 89% and resistant loading with increased PVR in 46% in patients with intermediate RV afterload (Figure 2). In patients with high afterload, 91% had markedly reduced PAC and 47% had markedly increased PVR. The agreement between the ESC/ERS 2022 classification (no PH, IpcPH, CpcPH) and the RV afterload score (low, intermediate, high) was moderate with 63% observer agreement and kappa 0.46 (95% CI: 0.39-0.54) (Supplemental Figure 1). Using the RV afterload score, there was a significant step up in the proportion of patients having signs of severely reduced cardiac function and pulmonary vascular dysfunction (peak maximum rate of oxygen consumption ≤14 mL/kg/min, systolic PA pressure ≥60 mm Hg, PA wedge pressure >15 mm Hg, PA effective elastance ≥1.3 mm Hg/mL, and mixed venous oxygen saturation <60%) between intermediate and high RV afterload (Figure 3A). Using the ESC/RES definition, overall, the step-up between IpcPH and CpcPH was smaller (systolic PA pressure, pulmonary artery effective elastance, mixed venous oxygen saturation) or not significant (peak maximum rate of oxygen consumption, PA wedge pressure) (Figure 3B).

### Grading severity of RV afterload

**Figure 4:**
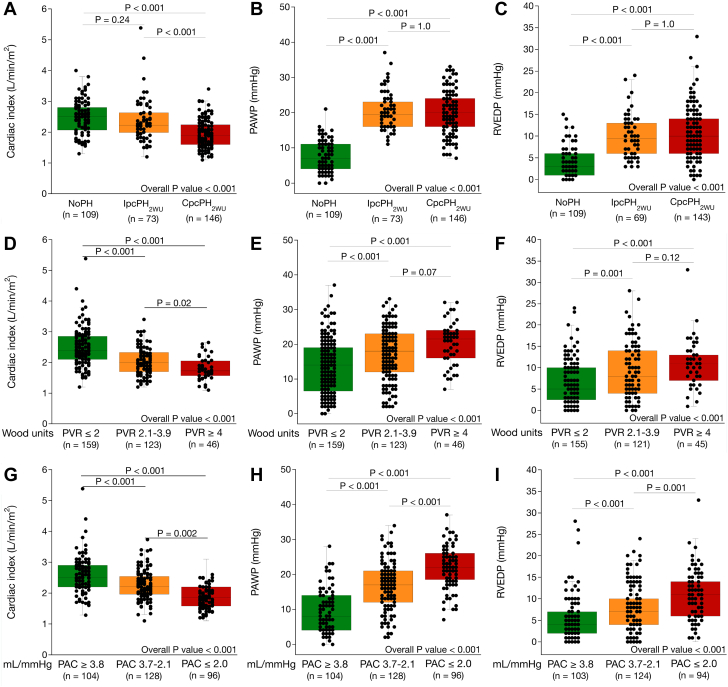
**Stepwise Severity Relation Using 2022 European Society of Cardiology/European Respiratory Society Phenotype, Pulmonary Vascular Resistance Intervals, or Pulmonary Artery Compliance Intervals** Boxplots showing the cardiac index (A, D, G), mean pulmonary artery pressure (B, E, H), and right ventricular end-diastolic pressure (C, F, I) in patients according to the 2022 European Society of Cardiology/European Respiratory Society phenotype (A to C), increments of pulmonary vascular resistance (D to F), and pulmonary artery compliance (G to I). CpcPH = combined pre- and postcapillary pulmonary hypertension; IpcPH = isolated postcapillary pulmonary hypertension; NoPH = no pulmonary hypertension; PAC = pulmonary artery compliance; PAWP = pulmonary artery wedge pressure; PVR = pulmonary vascular resistance; RVEDP = right ventricular end-diastolic pressure.

Using the 2022 ESC/ERS PH definition, 109 patients (33%) had no PH, 73 patients (22%) had IpcPH, and 146 patients (45%) had CpcPH. Patients with CpcPH had a lower working capacity compared with IpcPH, a similar degree of LV and RV backward failure but lower cardiac index (Table 2, Figures 4A to 4C).

**Table 2:** Clinical Characteristics, LV Function, RV Afterload, and RV Function in Different PH Subtypes According to the 2022 ESC/ERS Definition

	2022 ESC/ERS Definition			Overall *P* Value	Post-Hoc Analysis∗		
	NoPH (n = 109)	IpcPH (n = 73)	CpcPH (n = 146)		NoPH vs IpcPH	NoPH vs CpcPH	IpcPH vs CpcPH
Clinical							
Age (y)	54 ± 12	52 ± 15	54 ± 13	0.571	-	-	-
Male (%)	80	81	71	0.647	-	-	-
BMI (kg/m^2^)	27.2 ± 5.0	27.4 ± 4.4	26.7 ± 4.6	0.669	-	-	-
Months of follow-up	75 (13; 132)	11.4 (2; 98)	17.7 (4; 116)	**<0.001**	**0.001**	**0.014**	0.448
Censored due to							
Htx (%)	31	53	40	0.065	-	-	-
LVAD (%)	5	15	10	0.068	-	-	-
Mortality (%)	23	23	29	0.600	-	-	-
Laboratory							
Creatinine	100 (83; 136)	99 (84; 129)	101 (84; 126)	0.973	-	-	-
NT-proBNP	1,530 (647; 2,880)	3,380 (1,580; 5,728)	3,410 (1,946; 5,998)	**<0.001**	**<0.001**	**<0.001**	1.000
Functional capacity							
NYHA functional class III-IV (%)	58	79	84	**0.045**	**0.007**	**<0.001**	1.000
Peak workload (W)	87 (71; 122)	91 (74; 106)	73 (57; 94)	<0.001	1.000	**0.002**	**0.043**
Peak VO_2_ (mL/kg/min)	14.0 (11.3; 16.9)	13.6 (10.4; 16.2)	12.4 (10.3; 15.6)	0.063	-	-	-
LV/LA dimension and function							
LV ejection fraction (%)	27 (22; 35)	23 (19; 32)	23 (18; 28)	**<0.001**	**0.008**	**<0.001**	1.000
LV GLS (%)	−8 (−11; −7)	−6 (−8; −5)	−6 (−8; −5)	**<0.001**	**<0.001**	**<0.001**	1.000
LAVI (mL/m^2^)	40 (31; 53)	58 (44; 69)	52 (44; 65)	**<0.001**	**<0.001**	**<0.001**	1.000
MAP (mm Hg)	76 ± 14	75 ± 12	75 ± 12	0.697	-	-	-
PAWP (mm Hg)	7 (4; 11)	20 (16; 23)	20 (16; 24)	**<0.001**	**<0.001**	**<0.001**	1.000
Cardiac index (L/min/m^2^)	2.5 ± 0.5	2.4 ± 0.7	1.9 ± 0.5	**<0.001**	0.242	**<0.001**	**<0.001**
RV afterload							
mPAP (mm Hg)	15 ± 4	27 ± 5	33 ± 7	**<0.001**	**<0.001**	**<0.001**	**<0.001**
sPAP (mm Hg)	24 ± 5	40 ± 8	50 ± 13	**<0.001**	**<0.001**	**<0.001**	**<0.001**
mPAP-PAWP (mm Hg)	7 (6; 9)	7 (6; 9)	12 (9; 16)	**<0.001**	1.000	**<0.001**	**<0.001**
sPAP-dPAP (mm Hg)	17 ± 5	21 ± 7	28 ± 10	**<0.001**	**<0.001**	**<0.001**	**<0.001**
Ea (mm Hg/mL)	0.33 (0.27; 0.42)	0.68 (0.46; 0.82)	0.94 (0.73; 1.21)	**<0.001**	**<0.001**	**<0.001**	**<0.001**
PAC (mL/mm Hg)	4.4 (3.7; 5.7)	2.9 (2.3; 3.8)	2.0 (1.5; 2.5)	**<0.001**	**<0.001**	**<0.001**	**<0.001**
PVR (WU)	1.5 (1.1; 2.0)	1.5 (1.2; 1.8)	3.1 (2.4; 4.3)	**<0.001**	1.000	**<0.001**	**<0.001**
RV dimension and function							
RVDAi (cm^2^/m^2^)	10.9 ± 3.9	11.9 ± 2.9	12.6 ± 2.9	**<0.001**	0.055	**<0.001**	0.334
RA area (cm^2^)	19.9 ± 7.5	25.6 ± 7.4	24.4 ± 7.1	**<0.001**	**<0.001**	**<0.001**	0.788
TAPSE (mm)	16 ± 5	15 ± 5	14 ± 5	**0.030**	0.267	**0.027**	1.000
Fractional area change (%)	35 ± 11	28 ± 11	25 ± 10	**<0.001**	**0.001**	**<0.001**	0.187
S velocity (cm/s)	9 ± 3	9 ± 3	8 ± 2	**0.045**	1.000	0.096	0.06
RV free-wall strain (%)	−17 ± 7	−15 ± 6	−16 ± 6	0.141	-	-	-
TR grade ≥2 (n/%)	13	24	40	**<0.001**	0.144	**<0.001**	0.102
IVC collapsibility (%)	79 (55; 100)	47 (28; 77)	37 (23; 64)	**<0.001**	**<0.001**	<0.001	0.322
RVEDP (mm Hg)	3 (1; 6)	9 (6; 13)	10 (6; 14)	**<0.001**	**<0.001**	**<0.001**	1.000
RVSWI (g/m/beat/m^2^)	5.9 (4.3; 7.2)	8.2 (5.8; 10.5)	8.7 (6.6; 10.8)	**<0.001**	**<0.001**	**<0.001**	0.729

**Bold** indicate *P* < 0.05. Values are mean ± SD, median and IQR (25%; 75%), or percentage.

BMI = body mass index; CpcPH = combined pre- and postcapillary pulmonary hypertension; Ea = pulmonary artery effective elastance; ESC/ERS = European Society of Cardiology/European Respiratory Society; Htx = heart transplantation; IpcPH = isolated postcapillary pulmonary hypertension; IVC = internal vena cava; LA = left atrium; LAVI = left atrial volume indexed to body surface area; LV = left ventricle/ventricular; LVAD = LV assist device; LV GLS = LV global longitudinal strain; MAP = mean systemic arterial blood pressure; NoPH = no pulmonary hypertension; NYHA = New York Heart Association; NT-proBNP = N-terminal pro–B-type natriuretic peptide; PAWP = pulmonary artery wedge pressure; PAC = pulmonary artery compliance; PVR = pulmonary vascular resistance; RA area = right atrial area; RV = right ventricle/ventricular; RVEDP = right ventricular end-diastolic pressure; RVDAi = right ventricular diastolic area indexed to body surface area; RVSWI = right ventricular stroke work index; S velocity = tissue Doppler systolic velocity; sPAP-dPAP = pulse pressure; TAPSE = tricuspid annular plane systolic excursion; TR = tricuspid regurgitation; VO_2_ = maximum rate of oxygen consumption.

∗ Post hoc analysis significance values have been adjusted by the Bonferroni correction for multiple tests.

Using increments of PVR, 160 patients (49%) had PVR ≤2 WU indicating low resistant load, 123 patients (38%) had PVR 2.1 to 3.9 indicating intermediate resistant load, and 46 patients (14%) had PVR ≥4 WU indicating high resistant load. Patients with high resistive load had significantly more severe overall cardiac dysfunction compared with intermediate, with lower cardiac index, a tendency to have more severe backward failure with higher PA wedge pressure but similar degree of RV dysfunction assessed from RV end-diastolic pressure (Supplemental Table 2, Figures 4D to 4F).

Using increments of PAC, 104 patients (32%) had PAC ≥3.8 mL/mm Hg indicating low pulsatile load, 128 patients (39%) had PAC 3.7-2.0 mL/mm Hg indicating intermediate pulsatile load, and 96 patients (29%) had PAC ≤2.0 mL/mm Hg indicating high pulsatile load. Patients with high pulsatile load had compared with intermediate pulsatile load significantly more severe overall cardiac dysfunction with lower cardiac index, more severe backward failure with higher PA wedge pressure, and more pronounced RV dysfunction with higher RV end-diastolic pressure (Supplemental Table 3, Figures 4G to 4I).

**Figure 5:**
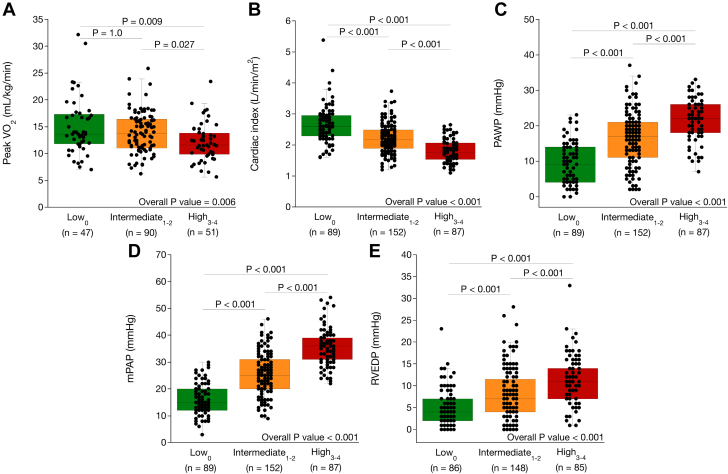
**Stepwise Severity Relation Using Right Ventricular Afterload Score** Boxplots showing peak maximum rate of oxygen consumption (VO_2_) (A), cardiac index (B), pulmonary artery wedge pressure (C), mean pulmonary artery pressure (D), and right ventricular end-diastolic pressure (E) in patients according to increments of right ventricular afterload. mPAP = mean pulmonary artery pressure; PAWP = pulmonary artery wedge pressure; RVEDP = right ventricular end-diastolic pressure.

Patients with high RV afterload score had compared with patients with intermediate RV afterload score (Table 3, Figure 5), more symptoms, lower working capacity, lower cardiac index, more severe LV backward failure with higher PA wedge pressure, and more severe RV dysfunction with higher proportion with ≥ moderate tricuspid regurgitation and higher RV end-diastolic pressure.

**Table 3:** Clinical Characteristics, LV Function, RV Afterload, and RV Function in Different PH Subtypes According to Increments of RV Afterload Score

	RV Afterload Score			Overall *P* Value	Post-Hoc analysis∗		
	Low (n = 89)	Intermediate (n = 152)	High (n = 87)		Low vs Intermediate	Low vs High	Intermediate vs High
Clinical							
Age (years)	52 ± 12	53 ± 14	56 ± 12	0.077	-	-	-
Male (%)	83	78	66	0.372	-	-	-
BMI (kg/m^2^)	27.9 ± 4.5	26.9 ± 4.8	26.2 ± 4.6	0.063	-	-	-
Months of follow-up	72.7 (11; 113)	23.8 (3.7; 130)	12 (3.3; 106)	**0.033**	0.309	**0.028**	0.586
Censored due to							
Htx (%)	36	43	39	0.701	-	-	-
LVAD (%)	2	11	14	**0.03**	**0.04**	**0.02**	1.000
Mortality (%)	20	25	28	**0.803**	-	-	-
Laboratory							
Creatinine	98 (83; 132)	100 (83; 126)	103 (85; 136)	0.583			
NT-proBNP	1,510 (615; 2,930)	3,060 (1,545; 5,515)	3,660 (1,800; 6,700)	**<0.001**	**<0.001**	**<0.001**	0.223
Functional capacity							
NYHA functional class III-IV (%)	59	75	87	0.095	-	-	-
Peak workload (W)	96 (78; 126)	83 (68; 106)	70 (54; 89)	**<0.001**	**0.047**	**<0.001**	**0.004**
Peak VO_2_ (mL/kg/min)	13.6 (11.8; 17.5)	13.7 (11; 16.5)	11.6 (9.8; 13.9)	<0.001	1.000	**0.009**	**0.027**
LV/LA dimension and function							
LV ejection fraction (%)	28 (22; 35)	24 (19; 31)	22 (19; 27)	**<0.001**	**0.002**	**<0.001**	0.339
LV GLS (%)	−8 (−11; −6)	−7 (−9; −5)	−6 (−8; −5)	**<0.001**	**0.028**	**0.001**	0.500
LAVI (mL/m^2^)	43 (33; 55)	53 (40;68)	52 (45; 62)	**<0.001**	**0.001**	**0.002**	1.000
MAP (mm Hg)	76 ± 13	75 ± 12	75 ± 12	0.598	-	-	-
PAWP (mm Hg)	9 (4; 14)	11 (17; 21)	22 (18; 26)	**<0.001**	**<0.001**	**<0.001**	**<0.001**
Cardiac index (L/min/m^2^)	2.6 ± 0.6	2.2 ± 0.5	1.8 ± 0.4	**<0.001**	**<0.001**	**<0.001**	**<0.001**
RV afterload							
mPAP (mm Hg)	16 ± 6	29 ± 8	36 ± 7	**<0.001**	**<0.001**	**<0.001**	**<0.001**
sPAP (mm Hg)	26 ± 7	39 ± 11	54 ± 13	**<0.001**	**<0.001**	**<0.001**	**<0.001**
mPAP-PAWP (mm Hg)	7 (6; 9)	9 (7; 11)	13 (9; 17)	**<0.001**	**<0.001**	**<0.001**	**<0.001**
sPAP-dPAP (mm Hg)	15 ± 5	22 ± 7	32 ± 11	**<0.001**	**<0.001**	**<0.001**	**<0.001**
Ea (mm Hg/mL)	0.31 (0.24; 0.39)	0.66 (0.48; 0.81)	1.1 (0.94; 1.46)	**<0.001**	**<0.001**	**<0.001**	**<0.001**
PAC (mL/mm Hg)	5.0 (4.3; 6.4)	2.8 (2.3; 3.3)	1.6 (1.2; 1.9)	**<0.001**	**<0.001**	**<0.001**	**<0.001**
PVR (WU)	1.4 (1.0; 1.6)	2.1 (1.5; 2.5)	4.1 (2.7; 5.1)	**<0.001**	**<0.001**	**<0.001**	**<0.001**
RV dimension and function							
RVDAi (cm^2^/m^2^)	10.7 ± 3.4	12.1 ± 3.4	12.8 ± 2.8	**<0.001**	**0.004**	**<0.001**	0.195
RA area (cm^2^)	21.3 ± 8.2	24.2 ± 7.7	23.6 ± 6.8	**0.004**	**0.003**	0.054	1.000
TAPSE (mm)	17 ± 5	15 ± 5	14 ± 5	**<0.001**	**0.009**	**0.002**	1.000
Fractional area change (%)	35 ± 12	29 ± 11	24 ± 9	**<0.001**	**0.001**	**<0.001**	**0.002**
S velocity (cm/s)	9 ± 3	8 ± 3	8 ± 2	**0.011**	0.162	**0.008**	0.463
RV free-wall strain (%)	−17 ± 6	−16 ± 7	−16 ± 6	0.475	-	-	-
TR grade ≥2 (n/%)	13	25	46	<0.001	0.105	**0.001**	**0.005**
IVC collapsibility (%)	67 (51; 100)	56 (29; 100)	33 (19; 53)	**<0.001**	**0.013**	**<0.001**	**<0.001**
RVEDP (mm Hg)	4 (4; 7)	7 (4; 12)	11 (7; 15)	**<0.001**	**<0.001**	**<0.001**	**<0.001**
RVSWI (g/m/beat/m^2^)	6.3 (4.6; 8.6)	7.2 (5.4; 9.6)	8.9 (6.4; 10.9)	**<0.001**	0.080	**<0.001**	0.058

**Bold** indicate *P* < 0.05. Values are mean ± SD, median and IQR (25%; 75%), or percentage.

Abbreviations as in Table 2.

∗ Post hoc analysis significance values have been adjusted by the Bonferroni correction for multiple tests.

### The PVR-PAC relation

**Figure 6:**
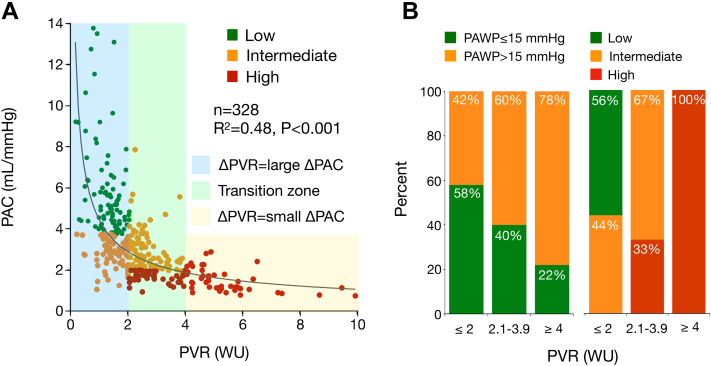
**Pulmonary Vascular Resistance, Pulmonary Artery Compliance, and Pulmonary Artery Wedge Pressure in Heart Failure** The relation between pulmonary vascular resistance and pulmonary artery compliance with the distribution of low, intermediate, and high right ventricular afterload score (A). The distribution of patients with increased pulmonary artery wedge pressure and normal, intermediate, and high right ventricular afterload according to increments of pulmonary vascular resistance (B). PAC = pulmonary artery compliance; PAWP = pulmonary artery wedge pressure; PVR = pulmonary vascular resistance.

The Central Illustration shows the overall relation between PAC and PVR as well as the impact of increased PA wedge pressure (left) and the distribution of RV afterload phenotypes (Figure 6, left). The best fit curve indicates a strong relation (R^2^ = 0.48) between PVR and PAC; however, the relation is influenced by PA wedge pressure. For any level of PVR, increased PA wedge pressure will lower the PAC. By visual inspection, the hyperbolic shape defines 3 different relations, the PVR ≤2 WU zone with large changes in PAC with small changes in PVR, the PVR ≥4 WU zone with small changes in PAC by large changes in PVR, and the transition zone PVR 2 to 4 WU with important changes in both PVR and PAC. The proportion with PA wedge pressure >15 mm Hg increased from 42% in patients with PVR ≤2 WU to 78% in patients with PVR ≥4 WU (Figure 6, right). In the PVR ≤2 WU zone, 44% had intermediate RV afterload score while in the PVR 2.1 to 2.9 WU zone, 67% had intermediate and 33% high RV afterload score. In the PVR ≥4 WU zone, all patients had a high RV afterload score.

### Clinical outcomes and prognostic value

**Figure 7:**
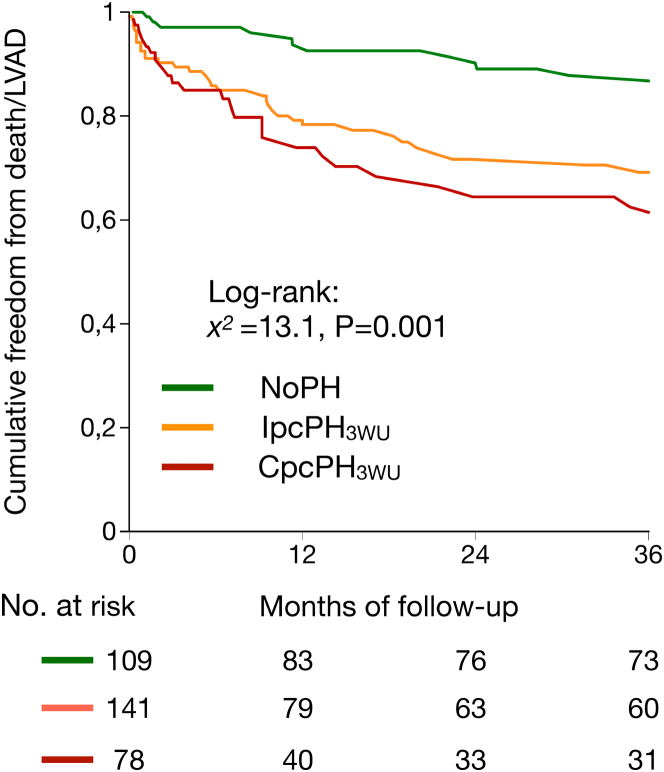
**Kaplan-Meier Curves for the Primary Endpoint** Kaplan-Meier curves for the primary endpoint of all-cause mortality and implantation of a left ventricular assist device in patients according to the 2018 WSPH classification. CpcPH = combined pre- and postcapillary pulmonary hypertension; IpcPH = isolated postcapillary pulmonary hypertension; NoPH = no pulmonary hypertension.

During a median follow-up time of 35.7 months (IQR: 4.4-120.2), 76 patients (23%) died, 31 patients (9%) received an LVAD, and 111 patients (34%) underwent heart transplantation. Within 36 months, 41 patients (13%) died, 26 patients (8%) received an LVAD, and 97 patients (30%) underwent heart transplantation. Figure 7 shows the Kaplan-Meier curves for the primary endpoint (survival free of death or LVAD implantation) in patients according to the 2018 WSPH (Figure 7), 2022 ESC/ERS (Central Illustration) hemodynamic definitions of PH, and increments in RV afterload score (Central Illustration). Both the 2018, WSPH 2018 and the 2022 ESC/ERS hemodynamic definitions, and the RV afterload score were associated with all-cause mortality and LVAD implantation, both unadjusted and adjusted for confounders (Table 4). The C-statistic was numerically higher for the RV afterload score than for the 2022 ESC/ERS definition (0.65 vs 0.62, *P* = 0.150). In contrast to the 2022 ESC/ERS definition, the RV afterload score showed worse survival with increasing score (Central Illustration, Table 4) and provided incremental prognostic value (*P* = 0.010). Compared with low RV afterload score, the HR for an intermediate score was 3.0, and 5.4 for the highest scores (Table 4), while HR were similar for IpcPH and CpcPH using the 2022 ESC/ERS definition (3.0 and 3.3, respectively). Increasing PVR and decreasing PAC were both associated with outcomes (HR [95% CI]: PVR [WU] 1.31 [1.15-1.51; PAC (mm Hg/mL): 0.67 (0.55-0.82)], and PAC provide incremental prognostic value to PVR alone, *P* = 0.002). Increasing PVR and decreasing PAC were both associated with outcomes (HR [95% CI]: PVR [WU] 1.31 [1.15-1.51; PAC (mm Hg/mL): 0.67 (0.55-0.82)], and PAC provide incremental prognostic value to PVR alone, *P* = 0.002). A model consisting of PVR and PAC as continuous variables showed numerically stronger discrimination than the RV Afterload score (C-statistic 0.68 vs 0.65; PAC HR: 0.78 [0.56-0.91]; PVR HR: 1.09 [0.90-1.32]) but the difference was not statistically significant (*P* = 0.06).

**Central Illustration:**
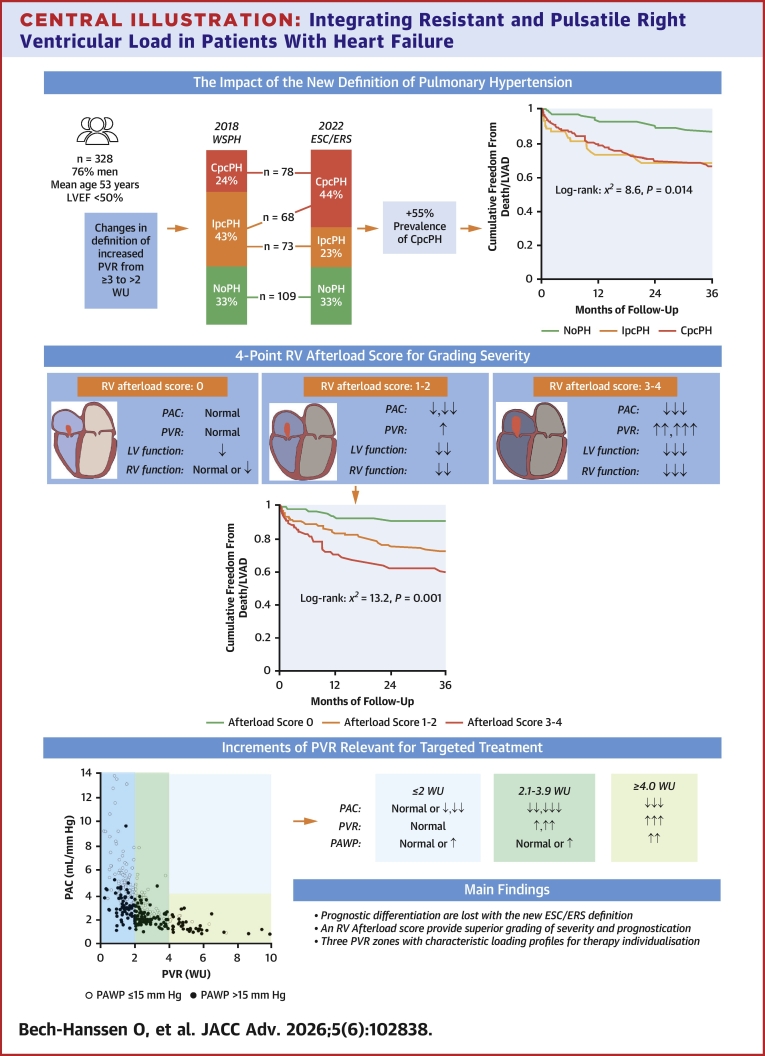
**Integrating Resistant and Pulsatile Right Ventricular Load in Patients With Heart Failure** We propose a novel right ventricular afterload score that integrates pulsatile and resistant components to grade right ventricular afterload severity in patients with left heart disease. This score improves severity stratification and prognostication compared with the current 2022 European Society of Cardiology/European Respiratory Society phenotypes and bundles properties of the pulmonary circulation with those of left ventricular function. The right ventricular afterload score may be useful for monitoring therapy response. In addition, the hyperbolic pulmonary vascular resistance–pulmonary artery compliance relation defines distinct pulmonary vascular resistance intervals with characteristic loading profiles that may support individualized therapy and in upcoming trials with new therapeutic targets. CpcPH = combined pre- and postcapillary pulmonary hypertension; ESC = European Society of Cardiology; ERS = European Respiratory Society; IpcPH = isolated postcapillary pulmonary hypertension; LV = left ventricle/ventricular; PAC = pulmonary artery compliance; PAWP = pulmonary artery wedge pressure; PVR = pulmonary vascular resistance; RV = right ventricle/ventricular; NoPH = no pulmonary hypertension; LVAD = LV assist device; LVEF = left ventricular ejection fraction.

**Table 4:** Death or LV Assist Device Within 36 Months

	Univariable Analysis		*C*	Multivariable Analysis	
	HR (95% CI)	*P* Value		HR (95% CI)	*P* Value
2018 WSPH					
NoPH	Reference		0.632		
IpcPH	2.94 (1.48-5.82)	**0.002**		2.72 (1.35-5.53)	**0.005**
CpcPH	3.83 (1.86-7.86)	**<0.001**		3.08 (1.46-6.49)	**0.003**
2022 ESC/ERS					
NoPH	Reference		0.617		
IpcPH	3.04 (1.41-6.55)	**0.005**		2.82 (1.29-6.13)	**0.009**
CpcPH	3.34 (1.71-6.51)	**<0.001**		2.88 (1.43-5.81)	**0.003**
RV afterload score					
Low	Reference		0.647		
Intermediate	3.04 (1.33-6.92)	**0.008**		2.71 (1.17-6.23)	**0.020**
High	5.35 (2.35-12.2)	**<0.001**		4.42 (1.86-10.6)	**<0.001**

**Bold** indicate *P* < 0.05. ∗Adjusted for age, sex, left ventricular ejection fraction, moderate/severe mitral regurgitation, and N-terminal pro–B-type natriuretic peptide.

Abbreviations as in Table 2.

Supplemental Figure 2 shows the Kaplan-Meier curves for the secondary endpoints (all-cause mortality and all-cause mortality/implantation of an LVAD/heart transplantation). As with the primary endpoint, according to the 2018 WSPH definition, patients with CpcPH showed the worst outcome (Supplemental Figures 2A and 2D). Similarly to the primary analysis, the RV afterload score, in contrast to the 2022 ESC/ERS definition, showed a stepwise increase in risk with increasing score (Supplemental Table 4, Supplemental Figure 2).

## Discussion

The main findings in the present study are: 1) as a consequence of the 2022 ESC/ERS PH hemodynamic definition, in patients with heart failure with reduced or midrange LV ejection fraction, lowering the PVR threshold from ≥3 to >2 WU, more than 50% of patients previously considered to have IpcPH were reclassified to CpcPH, and the primary outcome did not differ between patients with CpcPH compared with IpcPH; 2) a novel RV afterload score integrating both resistant and pulsatile load components provided superior grading of disease severity and prognosis. Increasing RV afterload score was associated with stepwise worsening of functional capacity, RV and LV function, and survival; and 3) the hyperbolic relationship between PVR and PAC allowed identification of 3 distinct PVR zones with characteristic loading profiles that might be helpful for individualization of heart failure therapy and selection of patients in upcoming trials with emerging treatment targets.

### The impact of the new PH definition

The revised thresholds for mean PA pressure and PVR in the recent PH hemodynamic definition represent the upper normal limits of these parameters,^11^ and large retrospective cohort studies has shown the prognostic relevance of mildly elevated PA pressure^12^ and PVR.^13^ The rationale for changing the hemodynamic definition of PH was to facilitate earlier detection of precapillary pulmonary vascular disease.^2^ However, in patients with PH due to left heart disease, constituting the most common PH subtype, the scenario is different and more challenging. Lowering the threshold for mean PA pressure and PVR markedly increases the proportion of patients considered to have PH and CpcPH, respectively. We can foresee a positive effect of considering patients with left heart disease having PH at an earlier stage because this might be a trigger for optimization of heart failure therapy including earlier intervention in heart valve disease. The pathophysiology of PH in patients with left heart disease is complex and therefore there is a need for a more diversified classification considering both resistant and pulsatile load. The prevalence of PH in patients with left heart disease will depend on the diagnostic method, cutoff values and the population studied. In the present study, 67% had PH (mean PA pressure>20 mm Hg) consistent with observational studies showing a prevalence 40% to 72%.^2^ Concerning CpcPH prevalence using the recently adopted PVR threshold >2 WU, only 1 prior study is available. In that prospective study, Fauvel et al.^14^ reported that the new definition increased the proportion of CpcPH from 46% to 73%. The corresponding result in our study was an increase from 24% to 44%. Including patients with mildly elevated PVR in the CpcPH phenotype has a diluting effect regarding severity profile because they have compared with patients with PVR >3 WU, less severe left heart disease, less increased RV afterload, and less severe RV dysfunction. In the study by Fauvel et al.^14^ CpcPH showed compared with IpcPH, the worst survival with separated Kaplan-Meier survival curves. The corresponding survival curves for the primary endpoint using the 2018 WSPH definition was not described. Importantly, their study population was markedly different from ours with heart failure with preserved ejection fraction in 42% vs exclusive heart failure with reduced ejection fraction in our study, and a primary endpoint including heart failure hospitalization. Of interest and in agreement with our study findings, Fauvel et al. also demonstrated the prognostic impact of increments of PVR (<2 WU, 2-5 WU, >5 WU) and importance of pulsatile load (PAC).

### The RV afterload score for grading severity

The importance of pulsatile loading as a determinant of RV afterload and prognosis has been highlighted by other investigators.^3^, ^4^, ^5^, ^6^^,^^15^ However, to the best of our knowledge this is the first study that describe a composite RV afterload score that integrates both resistant and pulsatile components. Incremental increases in the RV afterload score were associated with stepwise worsening *(P* < 0.001) of RV dysfunction (RV end-diastolic pressure), overall cardiac function (cardiac index), and LV dysfunction (PA wedge pressure). Notably, PAC showed a stronger and more consistent gradient across severity categories than PVR.

The pulsatile load is augmented by LV backward failure and therefore, PAC bundles the compliance properties of the pulmonary circulation with LV function. Dupont et al^4^ found the PAC and not PVR to be an independent predictor of all-cause mortality or heart transplantation. Still, severely increased PVR is a hallmark of the end stage in patients with left heart disease with therapeutical and prognostic implications. The RV afterload score allows grading of severity of both RV dysfunction and LV dysfunction and should be of interest for monitoring changes following treatment.

In patients planned for a LVAD, postoperative RV failure is frequently occurring with high morbidity and mortality. Existing risk models for prediction of postoperative RV failure include PA wedge pressure, right atrial pressure, cardiac index, and PVR but not PAC, as hemodynamic variables.^16^ The overall performance of these models is poor, and the clinical use remains limited. In this context, the proposed RV afterload score could be of interest as it integrates both resistant and pulsatile load.

### PVR increments relevant for targeted treatment strategies

The PVR and PAC are inversely related in a hyperbolic manner, and our data define 3 physiologically distinct PVR zones with potential therapeutical implications. The PVR ≤2 WU zone is characterized by large changes in PAC with small changes in PVR as the patient becomes decompensated with mildly elevated mean PA pressure, while in the PVR ≥4 WU zone as the disease and severity of PH augments, the change in PAC will be small (Central Illustration, Figure 6).^17^ The intermediate zone (PVR 2.1-3.9 WU) represents a transition phase in which both resistance and pulsatile load remain modifiable. The different mechanisms involved represent treatment targets starting with standard heart failure treatment that by lowering PA wedge pressure will enhance compliance and reduce the pulsatile load. The experience so far using pulmonary arterial hypertension specific treatment in patients with left heart disease and PH has been overall negative,^18^, ^19^, ^20^, ^21^, ^22^, ^23^, ^24^, ^25^ and therefore such treatment is not recommended in the 2022 ESC/ERS guidelines.^2^ The inclusion criteria in previous trials on pulmonary arterial hypertension specific treatment in patients with left heart disease and PH has been a certain level of PH,^18^^,^^20^^,^^21^^,^^23^^,^^26^ but only a few trials has required PVR >3 WU as well.^22^^,^^24^^,^^25^ Finding potential treatment targets require better understanding of the underlying pathophysiological mechanism in PH in left heart disease, accordingly proper selection of patients and refinement of classification.^27^ In this context, we propose, instead of 1 PVR threshold, to consider increments of PVR (≤2 WU, 2.1-3.9 WU, ≥4 WU) because they capture principally different PVR-PAC relations relevant for targeted treatment strategies.

### Study strengths and limitations

A total of 188 patients (57%) had assessment of functional capacity with an ergospirometry test.

Several limitations should be discussed. The present study is retrospective and from a single-center with most of the patients being males (77%) undergoing heart transplant workup, that indicate advanced heart failure with reduced ejection fraction. For the study objective, we needed right heart catheterization data and therefore the study population will not be representative for the overall heart failure population. In the overall heart failure population, heart failure with preserved ejection fraction is considered to be more prevalent than heart failure with reduced ejection fraction.^28^ Regardless of the PVR threshold used, CpcPH is a frequent finding in patients with heart failure with preserved ejection fraction.^14^^,^^29^ There is, however, less likely that the relation between increased PVR and measures of LV decompensation, RV dysfunction, or pulsatile load is different in patients with heart failure with preserved ejection fraction compared with heart failure with reduced ejection fraction. Still, our results need external validation in larger study samples and in patients with heart failure with preserved ejection fraction undergoing catheterization.

## Conclusions

We propose a novel RV afterload score that integrates pulsatile and resistant components to grade RV afterload severity in patients with left heart disease. This score improves severity stratification and prognostication compared with the current ESC/ERS PH phenotypes, and bundles properties of the pulmonary circulation with those of LV function. The RV afterload score may therefore be useful for monitoring therapy response. In addition, the hyperbolic PVR-PAC relation defines distinct PVR intervals with characteristic loading profiles that may support individualized therapy and in upcoming trials with new therapeutic targets.

## Funding support and author disclosures

The study was supported by grants from the 10.13039/501100003793Swedish Heart-Lung Foundation, Stockholm, Sweden (Project no. 20230379) and the Swedish state under the agreement between the Swedish government and the county councils, the ALF-agreement (grant no.: 76250). The authors have reported that they have no relationships relevant to the contents of this paper to disclose.
