# Supplementary material for: Integrating Resistant and Pulsatile Right Ventricular Load Improves Severity Assessment in Heart Failure Patients
Source: JACC Adv. 2026 Jun 17;5(6):102838. doi: 10.1016/j.jacadv.2026.102838 (PMC13308250; doi:10.1016/j.jacadv.2026.102838)
Supplement: Supplemental_Material [file mmc1.pdf]

Supplementary TABLE 1 RV Afterload Score

	Allocated points in score		
	0	1	2
PAC (mL/mmHg)	≥ 3.8	3.7 – 2.1	≤ 2.0
PVR (Wood units)	≤ 2	2.1-3.9	≥ 4

PAC, pulmonary artery compliance; PVR, pulmonary vascular resistance, RV, right ventricle.

Supplementary Table 2 Clinical Characteristics, LV function, RV Afterload and RV Function in Different PH Subtypes of RV Resistive Loading (PVR).

	Increments of PVR				*Post hoc analysis**		
	Low (n=159)	Intermediate (n=123)	High (n=46)	Overall P value	Low *vs* Intermediate	Low vs High	Intermediate *vs* High
Clinical							
Age (years)	52±13	54±13	56±12	0.380	-	-	-
Male gender (%)	82	72	65	0.447	-	-	-
BMI (kg/m^2)	27.3±4.7	27.0±4.7	26.0±4.5	0.260	-	-	-
Months of follow-up	57.6 (4.9; 114)	31.6 (5.7; 131)	7.2 (2.7; 85)	0.108	-	-	-
Censored due to							
Htx (%)	40	39	43	0.930	-	-	-
LVAD (%)	8	10	13	0.650	-	-	-
Mortality (%)	20	24	26	0.667	-	-	-
Laboratory							
Creatinine	100 (84;136)	100 (83;123)	101 (84;140)	0.628	-	-	-
NT-proBNP	2220 (964;4420)	2880 (1455;5790)	3450 (1680;5865)	**0.014**	0.074	**0.040**	1.000
Functional capacity							
NYHA class 3-4 (%)	67	79	85	0.363	-	-	-
Peak Workload (watt)	90 (72;111)	79 (63;103)	63 (51;82)	**<0.001**	0.055	**<0.001**	**0.026**

Peak VO_2 (mL/kg/min)	13.9 (11.1;16.8)	12.9 (11.0;16.2)	11.9 (9.7;14.6)	0.048	0.728	**0.045**	0.349
LV/LA dimension and function							
LVEF (%)	26 (20;34)	23 (18;29)	25 (20;29)	**0.041**	**0.039**	0.654	1.000
LV GLS (%)	-7 (-10;-6)	-7 (-9;-5)	-6 (-8;-5)	**0.100**	-	-	-
LAVI (mL/m^2)	47 (37;62)	52 (44;65)	51 (42;62)	**0.181**	-	-	-
MAP (mmHg)	75±12	76±12	74±14	**0.546**	-	-	-
PAWP (mmHg)	14 (6;19)	18 (12;23)	22 (16;24)	**<0.001**	**<0.001**	**<0.001**	0.072
Cardiac index (L/min/m^2)	2.5±0.6	2.0±0.4	1.8±0.4	**<0.001**	**<0.001**	**<0.001**	**0.018**
RV afterload							
mPAP (mmHg)	20±8	28±8	38±8	**<0.001**	**<0.001**	**<0.001**	**<0.001**
sPAP (mmHg)	31±10	42±12	58±14	**<0.001**	**<0.001**	**<0.001**	**<0.001**
mPAP-PAWP (mmHg)	7 (5; 8)	11 (8;13)	17 (15;19)	**<0.001**	**<0.001**	**<0.001**	**<0.001**
sPAP-dPAP (mmHg)	18 (14;22)	22 (18;29)	33 (29;41)	**<0.001**	**<0.001**	**<0.001**	**<0.001**
Ea (mmHg/mL)	0.42 (0.29;0.65)	0.77 (0.55;1.0)	1.3 (0.93;1.57)	**<0.001**	**<0.001**	**<0.001**	**<0.001**
PAC (mL/mmHg)	4.0 (2.9;5.2)	2.4 (1.9; 3.2)	1.4 (1.1; 1.8)	**<0.001**	**<0.001**	**<0.001**	**<0.001**
RV dimension and function							
RVDAi (cm^2/m^2)	11.3±3.3	12.3±3.4	13.0±2.9	**0.001**	**0.035**	**0.003**	0.514
RA area (cm^2)	22.9±8.0	23.8±7.1	23.3±7.6	0.418	-	-	-

TAPSE (mm)	16±5	15±5	14±5	0.100	-	-	-
FAC (%)	32±12	27±11	24±9	**<0.001**	**0.007**	**0.001**	0.572
S velocity (cm/s)	9±3	8±2	7±2	**0.011**	0.195	**0.016**	0.526
RV free-wall strain (%)	-16±6	-17±7	-14±6	0.118	**-**	**-**	**-**
TR≥ grade 2 (n/%)	18	32	43	**0.006**	**0.024**	**0.002**	**0.624**
IVC collapsibility (%)	61 (33;100)	43 (26;76)	36 (20;56)	**<0.001**	**0.031**	**<0.001**	0.151
RVEDP (mmHg)	5 (2;10)	8 (4;14)	11 (7;14)	**<0.001**	**0.001**	**<0.001**	0.116
RVSWI (g/m/beat/m^2)	6.4 (5.1;9.0)	7.6 (5.2;10.1)	9.3 (7.7;12.7)	**<0.001**	0.255	**0.001**	**0.002**

Bold indicate $P<0.05$. Values are mean ± SD, median and interquartile range (25%; 75%) or percentage. BMI = body mass index; Ea = pulmonary artery effective elastance; FAC = fractional area change; Htx = heart transplantation; IVC = internal vena cava; LAVI = left atrial volume indexed to body surface area; LV = left ventricle; LVAD = left ventricular assist device; LVEF = left ventricular ejection fraction; LV GLS = left ventricular global longitudinal strain; MAP = mean systemic arterial blood pressure; NYHA = New York Heart Association; NT-proBNP = N-terminal prohormone of brain natriuretic peptide; PAWP = pulmonary artery wedge pressure; RA area = right atrial area; RV = right ventricle; RVEDP = right ventricular end diastolic pressure; RVDAi = right ventricular diastolic area indexed to body surface area; RVSWI = right ventricular stroke work index; S velocity = tissue Doppler systolic velocity; TAPSE = tricuspid annular plane systolic excursion; TR = tricuspid regurgitation; VO_2 = maximum rate of oxygene consumption. *Post-hoc analysis significance values have been adjusted by the Bonferroni correction for multiple tests.

Supplementary TABLE 3 Clinical Characteristics, LV function, RV Afterload and RV Function in Different PH Subtypes of RV Pulsatile Loading (PAC).

	Increments of PAC				*Post hoc analysis**		
	Low (n=104)	Intermediate (n=128)	High (n=96)	Overall *P* value	Low *vs* Intermediate	Low vs High	Intermediate *vs* High
Clinical							
Age (years)	53±12	52±14	56±13	**0.026**	1.000	**0.047**	0.057
Male gender (%)	83	77	68	0.467	-	-	-
BMI (kg/m^2)	27.9±4.4	26.7±4.9	26.4±4.6	**0.034**	0.105	0.050	1.000
Months of follow-up	71.7 (11.3; 112)	34.4 (4.4; 131)	9.6 (3.0; 106)	**0.009**	0.934	**0.008**	0.094
Censored due to							
Htx (%)	35	44	41	0.547	-	-	-
LVAD (%)	3	11	15	**0.022**	0.067	**0.012**	1.000
Mortality (%)	20	24	24	0.798	-	-	-
Laboratory							
Creatinine	98 (82;131)	103 (84;131)	101 (86;131)	0.818	-	-	-
NT-proBNP	1195 (614;2893)	3115 (1623;5442)	4215 (2015;6810)	**<0.001**	**<0.001**	**<0.001**	0.204
Functional capacity							
NYHA class 3-4 (%)	63	73	91	0.080	-	-	-
Peak Workload (watt)	94 (76;124)	83 (68;98)	70 (54;92)	**<0.001**	0.066	**<0.001**	**0.043**

Peak VO_2 (mL/kg/min)	13.6 (11.8;17.1)	13.6 (11;16.4)	11.6 (9.9;14.6)	**0.017**	1.000	**0.022**	0.075
LV/LA dimension and function							
LVEF (%)	28 (22;35)	24 (19;31)	22 (18;27)	**<0.001**	**0.005**	**<0.001**	0.066
LV GLS (%)	-8 (-11;-6)	-7 (-9;-5)	-6 (-8;-4)	**<0.001**	0.121	**<0.001**	**0.010**
LAVI (mL/m^2)	42 (33;54)	54 (42;69)	52 (45;62)	**<0.001**	**<0.001**	**<0.001**	1.000
MAP (mmHg)	78±14	73±11	76±12	**0.023**	**0.025**	1.000	0.203
PAWP (mmHg)	8 (4;14)	17 (12;21)	22 (18;26)	**<0.001**	**<0.001**	**<0.001**	**<0.001**
Cardiac index (L/min/m^2)	2.6±0.6	2.2±0.5	1.8±0.4	**<0.001**	**<0.001**	**<0.001**	**<0.001**
RV afterload							
mPAP (mmHg)	17±6	26±8	35±7	**<0.001**	**<0.001**	**<0.001**	**<0.001**
sPAP (mmHg)	26±7	40±11	53±13	**<0.001**	**<0.001**	**<0.001**	**<0.001**
mPAP-PAWP (mmHg)	7 (6; 10)	9 (7;12)	12 (8;16)	**<0.001**	**<0.001**	**<0.001**	**0.001**
sPAP-dPAP (mmHg)	15±5	23±7	31±10	**<0.001**	**<0.001**	**<0.001**	**<0.001**
Ea (mmHg/mL)	0.32 (0.26;0.40)	0.66 (0.50;0.78)	1.1 (0.95;1.44)	**<0.001**	**<0.001**	**<0.001**	**<0.001**
PVR (WU)	1.4 (1.1;1.8)	2.1 (1.5; 2.6)	3.6 (2.4; 4.8)	**<0.001**	**<0.001**	**<0.001**	**<0.001**
RV dimension and function							
RVDAi (cm^2/m^2)	11.1±3.9	11.9±3.1	12.8±2.8	**<0.001**	0.061	**<0.001**	**0.039**
RA area (cm^2)	21.3±7.9	24.4±7.9	23.8±6.6	**0.002**	**0.002**	**0.015**	1.000

TAPSE (mm)	17±5	15±5	14±5	**<0.001**	0.090	**<0.001**	0.145
FAC (%)	34±12	29±11	24±8	**<0.001**	**0.006**	**<0.001**	**0.001**
S velocity (cm/s)	9±3	8±2	8±3	**0.090**	**-**	**-**	**-**
RV free-wall strain (%)	-17±7	-16±6	-15±6	0.078	**-**	**-**	**-**
TR≥ grade 2 (n/%)	13	28	39	**0.003**	**0.029**	**<0.001**	0.342
IVC collapsibility (%)	67 (51;100)	56 (28;97)	33 (18;53)	<0.001	**0.002**	**<0.001**	**0.001**
RVEDP (mmHg)	4 (2;7)	7 (4;12)	11 (7;14)	**<0.001**	<0.001	<0.001	0.001
RVSWI (g/m/beat/m^2)	6.3 (4.4;8.2)	7.8 (5.6;9.6)	8.3 (5.9;10.3)	**<0.001**	0.001	0.001	1.000

Bold indicate P<0.05. Values are mean ± SD, median and interquartile range (25%; 75%) or percentage. BMI = body mass index; Ea = pulmonary artery effective elastance; FAC = fractional area change; Htx = heart transplantation; IVC = internal vena cava; LAVI = left atrial volume indexed to body surface area; LV = left ventricle; LVAD = left ventricular assist device; LVEF = left ventricular ejection fraction; LV GLS = left ventricular global longitudinal strain; MAP = mean systemic arterial blood pressure; NYHA = New York Heart Association; NT-proBNP = N-terminal prohormone of brain natriuretic peptide; PAWP = pulmonary artery wedge pressure; RA area = right atrial area; RV = right ventricle; RVEDP = right ventricular end diastolic pressure; RVDAi = right ventricular diastolic area indexed to body surface area; RVSWI = right ventricular stroke work index; S velocity = tissue Doppler systolic velocity; TAPSE = tricuspid annular plane systolic excursion; TR = tricuspid regurgitation; VO_2 = maximum rate of oxygene consumption. *Post-hoc analysis significance values have been adjusted by the Bonferroni correction for multiple tests.

Supplementary Table 4 Death or LVAD with Full Follow-up Time

	Univariable analysis			Multivariable analysis*	
	Hazard Ratio (95% CI)	*P*	C	Hazard Ratio (95% CI)	*P*
**2018 WSPH**					
NoPH	Reference				
IpcPH	2.94 (1.48 – 5.82)	**0.002**	0.608	1.57 (0.97 – 2.54)	0.064
CpcPH	3.83 (1.86 – 7.86)	**<0.001**		2.00 (1.20 – 3.33)	**0.008**
**2022 ESC/ERS**					
NoPH	Reference				
IpcPH	1.92 (1.13 – 3.24)	**0.015**	0.596	1.82 (1.07 – 3.11)	**0.029**
CpcPH	1.82 (1.17 – 2.83)	**0.008**		1.69 (1.04 – 2.71)	**0.01**
**RV afterload score**					
Low	Reference				
Intermediate	1.81 (1.08 –3.03)	**0.024**	0.608	1.77 (1.04 – 3.00)	**0.036**
High	2.60 (1.52 –4.44)	**<0.001**		2.36 (1.31 – 4.24)	**0.004**

Bold indicate P<0.05. * Adjusted for age, sex, left ventricular ejection fraction, moderate/severe mitral regurgitation, and NT-proBNP. CI, confidence intervals; CpcPH, combined pre- and postcapillary pulmonary hypertension; IpcPH, isolated postcapillary pulmonary hypertension; NoPH, no pulmonary hypertension; WSPH, World Symposium on Pulmonary Hypertension.

PH phenotype by ESC/ERS 2022

RV afterload score	NoPH	IpcPH	CpcPH
Low	68	21	0
Intermediate	41	52	59
High	0	0	87

Kappa 0.46 (95% CI 0.39-0.54)
Observer agreement 63%

SUPPLEMENTARY FIGURE 1 Agreement between 2022 ESC/ERS Phenotype and RV Afterload Score

Cross tabs that show the agreement between 2022 ESC/ERS PH phenotypes and increments of the RV afterload score.

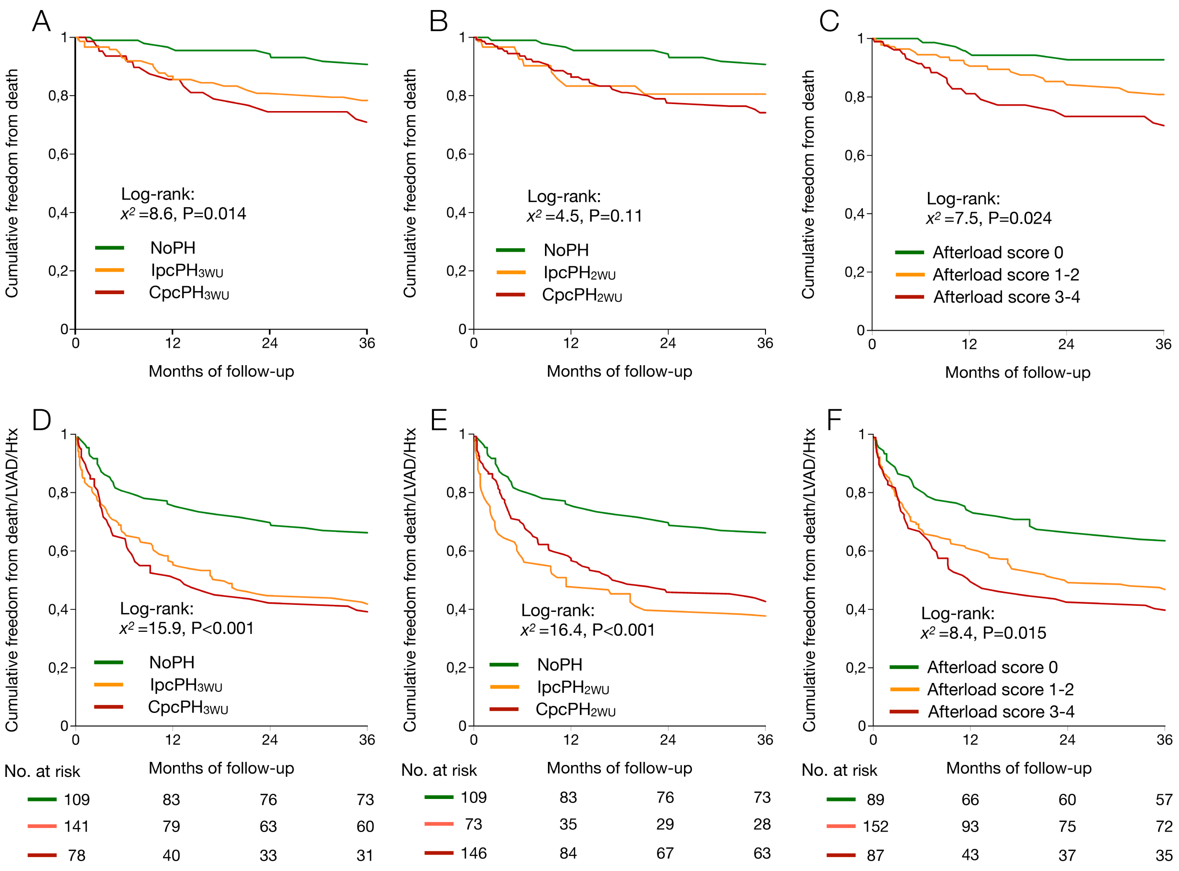

SUPPLEMENTARY FIGURE 2 Kaplan-Meier Curves for the Secondary Endpoints Kaplan-Meier curves for the secondary endpoints of all-cause mortality (A-C) and all-cause mortality, implantation of a left ventricular assist device or heart transplantation (D-F) in patients according to the 2018 WSPH classification (A, D), the 2022 ESC/ERS classification (B, E) and the RV afterload score (C, F).
